# Presumed Hippocampal Endometriosis Presenting as Catamenial Epilepsy: Diagnostic Challenges, MRI Follow-Up, and Comparative Review of Reported Cerebral Endometriosis Case

**DOI:** 10.3390/jcm15187062

**Published:** 2026-09-11

**Authors:** Mihaela-Camelia Tîrnovanu, Ștefan-Dragoș Tîrnovanu, Elena Cojocaru, Monica Holicov, Vlad-Constantin Donica, Roxana-Ana Covali, Awad Dmour, Norin Forna, Paul-Dan Sîrbu, Alin Ciubotaru

**Affiliations:** 1 Grigore T. Popa University of Medicine and Pharmacy Iași, 700115 Iași, Romania; mihaela.tirnovanu@umfiasi.ro (M.-C.T.); elena2.cojocaru@umfiasi.ro (E.C.); monica.holicov@umfiasi.ro (M.H.); vlad-constantin.donica@d.umfiasi.ro (V.-C.D.); ana.covali@umfiasi.ro (R.-A.C.); dmour-awad@umfiasi.ro (A.D.); norin.forna@umfiasi.ro (N.F.); paul.sirbu@umfiasi.ro (P.-D.S.); alinciubotaru94@yahoo.com (A.C.); 2 Cuza Vodă Women’s Clinical Hospital Iași, 700038 Iași, Romania

**Keywords:** cerebral endometriosis, catamenial epilepsy, hippocampal lesion, extrapelvic endometriosis, magnetic resonance spectroscropy, volumetric analysis, dienogest

## Abstract

**Background:** Cerebral endometriosis is an exceptionally rare manifestation of extrapelvic endometriosis and remains a diagnostic challenge because its clinical and imaging features may mimic more common neurological, inflammatory, vascular, or neoplastic disorders. Catamenial neurological symptoms may provide an important diagnostic clue, but histological confirmation is rarely available. **Case presentation:** We report the case of a 48-year-old woman with epilepsy beginning at 41 years of age, associated with short-term memory loss and a catamenial pattern of seizures. She had no typical symptoms of pelvic endometriosis, including dysmenorrhea, dyspareunia, or cyclic pelvic pain. Neuropsychological test for memory had normal scores. Brain magnetic resonance imaging (MRI) revealed a right hippocampal lesion with T2/FLAIR hyperintensity and later pseudonodular contrast enhancement, raising suspicion of focal cortical dysplasia, infection, tumor infiltration, or a hippocampal tumor. Magnetic resonance spectroscopy (MRS) was inconclusive and did not provide clear support for tumor-like infiltration. Because of the temporal association between symptoms and menstruation, cerebral endometriosis was considered. The patient received hormonal therapy with progestins, including desogestrel and dienogest, after which antiepileptic therapy was discontinued. Neurological symptoms resolved completely. Follow-up MRI demonstrated marked regression or disappearance of the previously described right hippocampal hyperintense lesion, with preserved bilateral hippocampal morphology. **Conclusions:** This case highlights the importance of considering cerebral endometriosis in women of reproductive or perimenopausal age presenting with catamenial epilepsy and unexplained intracranial lesions, even in the absence of pelvic endometriosis symptoms. Recognition of menstrual cyclicity, careful neuroimaging follow-up, and multidisciplinary evaluation may support diagnosis and guide individualized treatment. The favorable clinical and radiological response to progestin therapy in this case adds to the limited evidence regarding conservative management of presumed cerebral endometriosis. Cerebral endometriosis is a very rare condition, and only eight case reports were found in the literature.

## 1. Introduction

Endometriosis (EM) is an estrogen-dependent, chronic inflammatory disease de-fined by the presence of endometrial glands and/or stroma outside the uterine cavity. It primarily affects women of reproductive age and is one of the most common benign gynecological disorders after uterine fibroids. Because of its heterogeneous clinical presentation and ability to involve multiple anatomical sites, EM is sometimes referred to as the “chameleon of gynecology.” Although the disease most commonly affects pelvic structures, extrapelvic EM has been reported in several distant organs, including the pleura, lungs, diaphragm, kidneys, spleen, gastrointestinal tract, eyes (cyclical haemolacria, eye pain, vision changes, and light sensitivity during menstruation), surgical scars, and, exceptionally, the central nervous system [1]. The high recurrence rate and the involvement of such a large number of different organs and systems support the idea that endometriosis is a systemic condition [2]. The endometrium is a remarkable tissue with distinctive biological properties. Its ability to implant at ectopic sites while retaining functional responsiveness, particularly to hormonal stimulation, is a striking feature of the disease [3].

The pathogenesis of distant extrapelvic endometriosis remains incompletely understood. Several mechanisms have been proposed, including hematogenous or lymphatic dissemination of endometrial cells, coelomic metaplasia, stem or progenitor cell involvement, and altered immune surveillance. Hormonal sensitivity, chronic inflammation, impaired natural killer cell activity, and genetic or epigenetic susceptibility may also contribute to lesion survival and progression. However, the mechanisms by which endometrial-like tissue reaches and persists within the brain remain largely speculative because of the rarity of reported cases.

Cerebral EM is an exceptionally rare manifestation of extrapelvic EM, characterized by the presence or presumed presence of endometrial-like tissue within intracranial structures. Reported locations include the cerebellum, frontal lobe, and other cerebral regions [4]. Clinical manifestations vary according to lesion location and may include a range of neurological symptoms such as headache, seizures, focal neurological deficits (weakness, sensory disturbances, and paralysis), sensory or motor disturbances, visual or speech impairment, cognitive impairments (memory and concentration difficulties), or loss of consciousness. Psychiatric symptoms associated with EM in the brain may include behavior changes (heightened irritability, impulsiveness, and differences in social interactions), mood swings, depression, mood disturbances, and anxiety. In some cases, symptoms may fluctuate with the menstrual cycle, providing an important diagnostic clue. Nevertheless, the presentation is often nonspecific and may mimic more common neurological, vascular, inflammatory, infectious, or neoplastic conditions.

Catamenial epilepsy is characterized by a cyclical increase in seizure frequency during specific phases of the menstrual cycle and is generally defined by an approximately twofold increase in seizure occurrence during a hormonally vulnerable phase [5,6]. Although prevalence estimates vary according to the diagnostic criteria and study population, catamenial patterns have been identified in approximately one-third to one-half of women of reproductive age with epilepsy [7]. Fluctuations in ovarian steroid hormones are considered central to its pathophysiology: estrogens generally promote neuronal excitability and may exert proconvulsant effects, whereas progesterone and its neuroactive metabolites, particularly allopregnanolone, enhance γ-aminobutyric acid-mediated inhibition and generally exert anticonvulsant effects [8]. Three principal patterns have been described: the perimenstrual pattern (C1), characterized by seizure exacerbation from approximately 3 days before to 3 days after the onset of menstruation and associated predominantly with the abrupt premenstrual decline in progesterone; the periovulatory pattern (C2), associated with the mid-cycle estrogen surge; and the inadequate luteal-phase pattern (C3), observed during anovulatory or inadequate luteal-phase cycles with insufficient progesterone secretion [5,6].

Diagnosis is challenging and usually requires a combination of detailed medical history, assessment of symptom cyclicity, neurological examination, and neuroimaging. MRI is the preferred imaging modality for evaluating suspected intracranial lesions because it provides superior soft-tissue characterization and may identify hemorrhagic components or gliotic changes. On an MRI scan, cerebral EM typically appears as a cystic mass with signs of recurrent intracystic hemorrhage. They strongly resemble pelvic endometriomas, exhibiting the “T2 shadowing sign,” in which the cyst shows low signal intensity on both T1- and T2-weighted sequences due to old blood products [9]. However, imaging findings are not pathognomonic, and cerebral EM may resemble other cystic, hemorrhagic, inflammatory, or neoplastic brain lesions [10]. In selected cases, definitive diagnosis may require surgical excision or biopsy with histopathological confirmation.

There is no standardized treatment strategy for cerebral EM because of the very small number of documented cases. Management must therefore be individualized according to symptom severity, lesion location, radiological features, reproductive considerations, and surgical risk. Therapeutic options may include hormonal suppression, symptomatic treatment, neurosurgical intervention, or a combination of these approaches.

Among the hormonal treatments used for EM, progestins exert their principal effects through progesterone receptors, inducing decidualization and subsequent atrophy of eutopic endometrial and ectopic endometriotic tissue. Dienogest is a synthetic progestin widely used for the long-term treatment of EM. In addition to its direct anti-proliferative effects on endometriotic cells, dienogest moderately suppresses pituitary gonadotropin secretion and lowers endogenous estradiol concentrations, thereby limiting estrogen-dependent lesion activity [11]. It may also exert anti-inflammatory and antiangiogenic effects [12] and modulate molecular pathways involving aromatase and matrix metalloproteinases [13]. Desogestrel, generally administered as a progestin-only contraceptive, primarily suppresses the hypothalamic–pituitary–ovarian axis and inhibits ovulation by maintaining relatively stable progestogenic activity. This reduces ovarian estrogen production and may consequently suppress the stimulation and activity of endometriotic lesions [14].

Given the complexity of cerebral EM, multidisciplinary care involving gynecology, neurology, neurosurgery, radiology, pathology, and, when necessary, psychiatry or pain specialists is essential.

Cerebral EM remains an extremely rare and diagnostically challenging condition, with only a limited number of cases described in the literature. We present this case to contribute to the current understanding of this unusual manifestation of EM and to highlight the importance of considering EM in the differential diagnosis of intracranial lesions in women of reproductive age, particularly when neurological symptoms exhibit menstrual cyclicity.

## 2. Case Presentation

We present the case of a 48-year-old woman with epilepsy that began at the age of 41 and was associated with episodes of short-term memory loss. Menarche occurred at the age of 13. She reported regular menstrual cycles with normal menstrual flow. Her obstetric history included six elective abortions and one cesarean section at the age of 31. Her past medical history was otherwise unremarkable. Notably, she denied symptoms suggestive of pelvic endometriosis, including dysmenorrhea, dyspareunia, and cyclic abdominal or pelvic pain. In 2020, the patient underwent a contrast-enhanced abdominopelvic CT scan, which did not reveal pelvic endometriotic lesions. There was no first-degree family history of epilepsy.

The episodes were characterized by impaired or complete loss of awareness, behavioral arrest with staring, and postictal confusion. Following the initial neurological consultation, the patient maintained a combined seizure–menstrual diary for six months. The diary documented that the seizures occurred exclusively during menstrual bleeding, supporting a close temporal relationship with the menstrual cycle. A routine interictal electroencephalogram (EEG) was performed and showed no epileptiform discharges or other pathological abnormalities. Video-EEG monitoring and ictal EEG recording were not performed. Based on the temporal relationship between seizure exacerbations and menstruation, the patient’s clinical presentation was consistent with the perimenstrual (C1) pattern of catamenial epilepsy.

The patient was referred to a psychologist to evaluate her short-term memory problems. Common screening tools for memory and cognition were used, since she does not report serious short-term memory problems. The Mini-Cog test score was 5, the Mini-Mental State Examination (MMSE) had a score of 28, and the Montreal Cognitive Assessment (MoCA) score was 28. All these tests yielded normal results.

**Figure 1:**
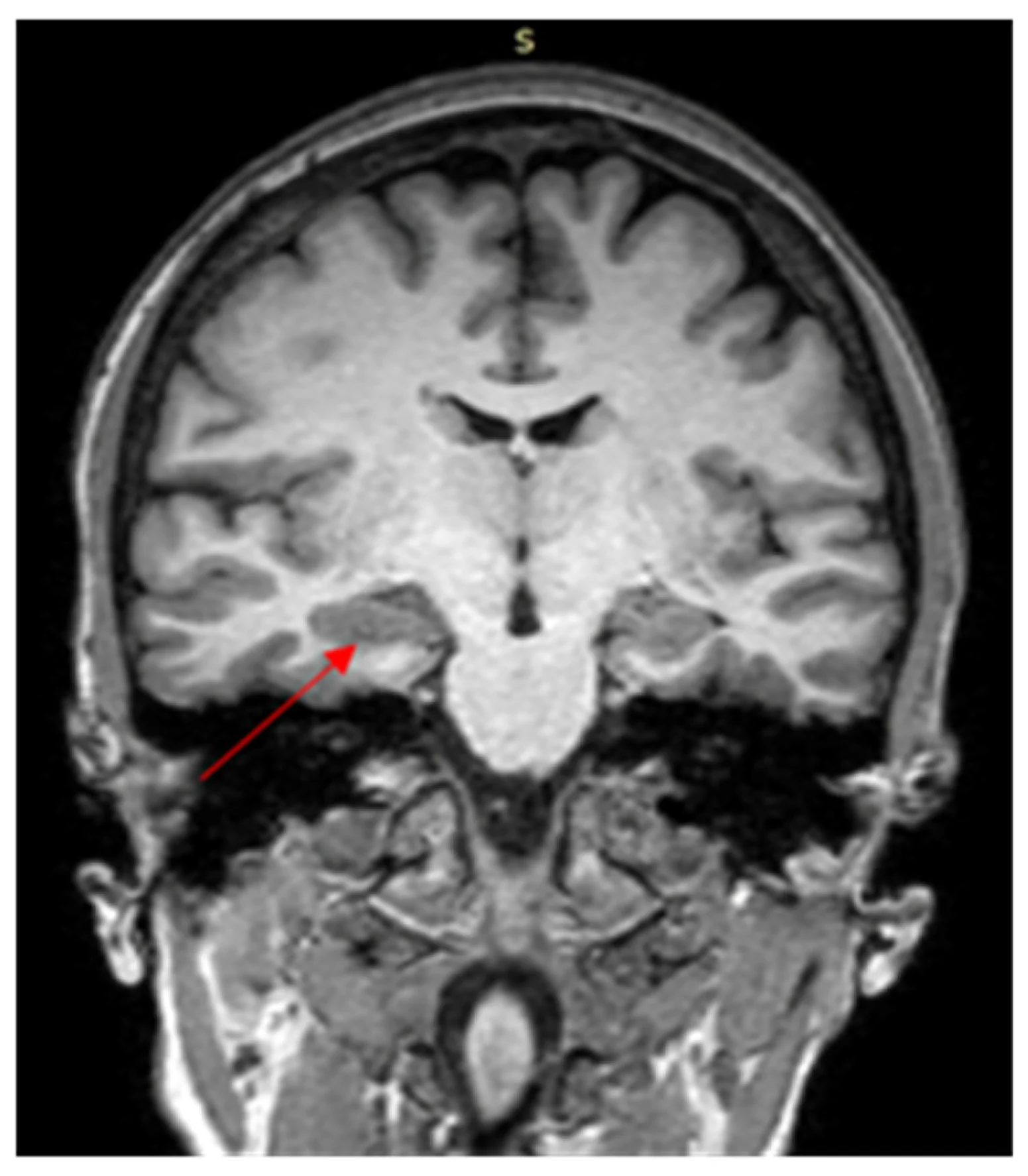
Whole-brain coronal T2-FLAIR MRI demonstrating the right hippocampal lesion (arrow).

The first contrast-enhanced brain MRI, performed in August 2019, revealed a diffuse lesion involving the right hippocampal cortico-subcortical region, including the head, body, and tail of the hippocampus (Figure 1). The lesion was mildly hypointense on T1-weighted images and hyperintense on T2-weighted and fluid-attenuated inversion recovery (FLAIR) sequences. No diffusion restriction or contrast enhancement was observed. The lesion was associated with cortical thickening and an edematous appearance of the adjacent gyri. Mild asymmetry of the cerebellar tonsils was also noted. Based on these imaging findings, the radiological differential diagnosis included focal cortical dysplasia, an infectious process, and tumor infiltration.

Short description of Figure 1:

- The image represents a coronal section through the medial temporal lobes, where the hippocampi, lateral ventricles, and medial temporal structures are visible.
- At the level of the right hippocampus (left side of the image), there is a focal hyperintense area on the FLAIR sequence, showing increased signal compared with the surrounding brain parenchyma.
- The finding suggests a focal lesion in the right hippocampal region.
- The contralateral hippocampus appears relatively normal in signal intensity and morphology.
- In this section, there is no significant midline shift or major mass effect.

Six months after symptom onset, treatment with levetiracetam 500 mg twice daily was initiated. After six months of therapy, epileptic seizures persisted; therefore, the antiepileptic regimen was modified, and lamotrigine (Lamictal) was introduced. For three months, the patient received combination therapy with levetiracetam and lamotrigine, with gradual titration of lamotrigine up to 100 mg/day. Levetiracetam was subsequently discontinued, and lamotrigine monotherapy continued for another three months. However, seizure frequency increased during lamotrigine monotherapy, and the patient was switched back to levetiracetam.

**Figure 2:**
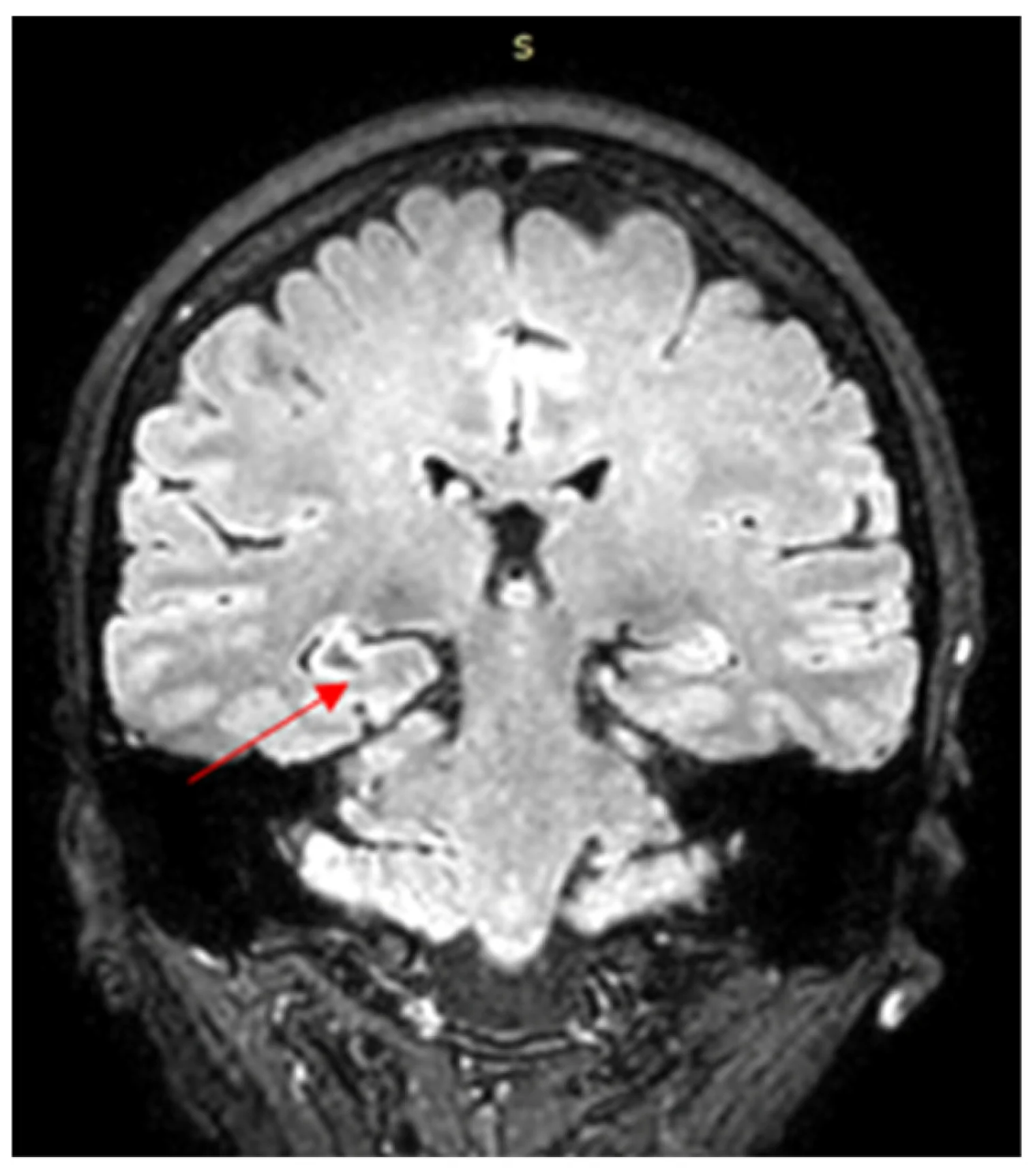
Whole-brain coronal MRI demonstrating the right hippocampal lesion (arrow). The image highlights the location and morphology of the lesion within the medial temporal lobe.

A follow-up brain MRI was performed six months after the initial examination, in 2020 (Figure 2). The examination revealed an 11 × 10 mm pseudonodular area of contrast enhancement in the posterior portion of the right hippocampus. A nodular T2-hypointense area was also identified in the same region, raising the possibility of calcification or hemosiderin deposition. Based on these imaging findings, the radiological impression favored a hippocampal tumor. However, given the presence of T1-hyperintense and T2-hypointense signal characteristics suggestive of hemorrhagic content, cerebral endometriosis was also considered in the differential diagnosis.

Short interpretation of Figure 2:

- This image shows a coronal section through the medial temporal lobes, including the hippocampi, temporal horns of the lateral ventricles, thalami, and surrounding temporal lobe structures.
- The cerebrospinal fluid in the lateral ventricles and temporal horns appears hyperintense, confirming a T2-weighted sequence.
- At the level of the right hippocampus, there is a subtle focal hyperintense signal with slight structural irregularity, compared with the contralateral side.
- The finding may correspond to a focal lesion involving the right hippocampal formation.
- The left hippocampus shows relatively preserved morphology and signal intensity.

The patient also underwent magnetic resonance spectroscopy (MRS). N-acetylaspartate (NAA), a neuronal marker that may decrease in conditions associated with neuronal loss or dysfunction, was reduced on the right side compared with the contralateral cerebral parenchyma. At long echo times, the NAA peak was not detectable on the right side, whereas it remained detectable contralaterally. Choline (Cho) and creatine (Cr) peaks were also analyzed. The Cho/NAA ratio was 0.8, slightly above the reported normal value of 0.6, while the Cho/Cr ratio was 0.9, close to the normal value of 1. No lipid–macromolecule or lactate peaks were detected. Overall, the MRS findings were inconclusive and did not provide clear evidence of tumor-like infiltration.

A treatment with progestins was initiated from the first visit to the gynecologist in 2021, when the cyclic appearance of epileptic seizures suggested cerebral EM. Between 2021 and 2025, the patient took hormonal treatment with dienogest 2 mg/day, alternated with Cerazette (desogestrel 75 μg/day) in six-month cycles. Levetiracetam was administered concomitantly for only three months. Subsequently, the patient continued treatment with progestin therapy for suspected cerebral EM and was also prescribed zopiclone 7.5 mg in the evening for severe insomnia. During this period, antiepileptic therapy was completely discontinued. Following progestin therapy, the patient reported complete resolution of neurological symptoms.

**Figure 3:**
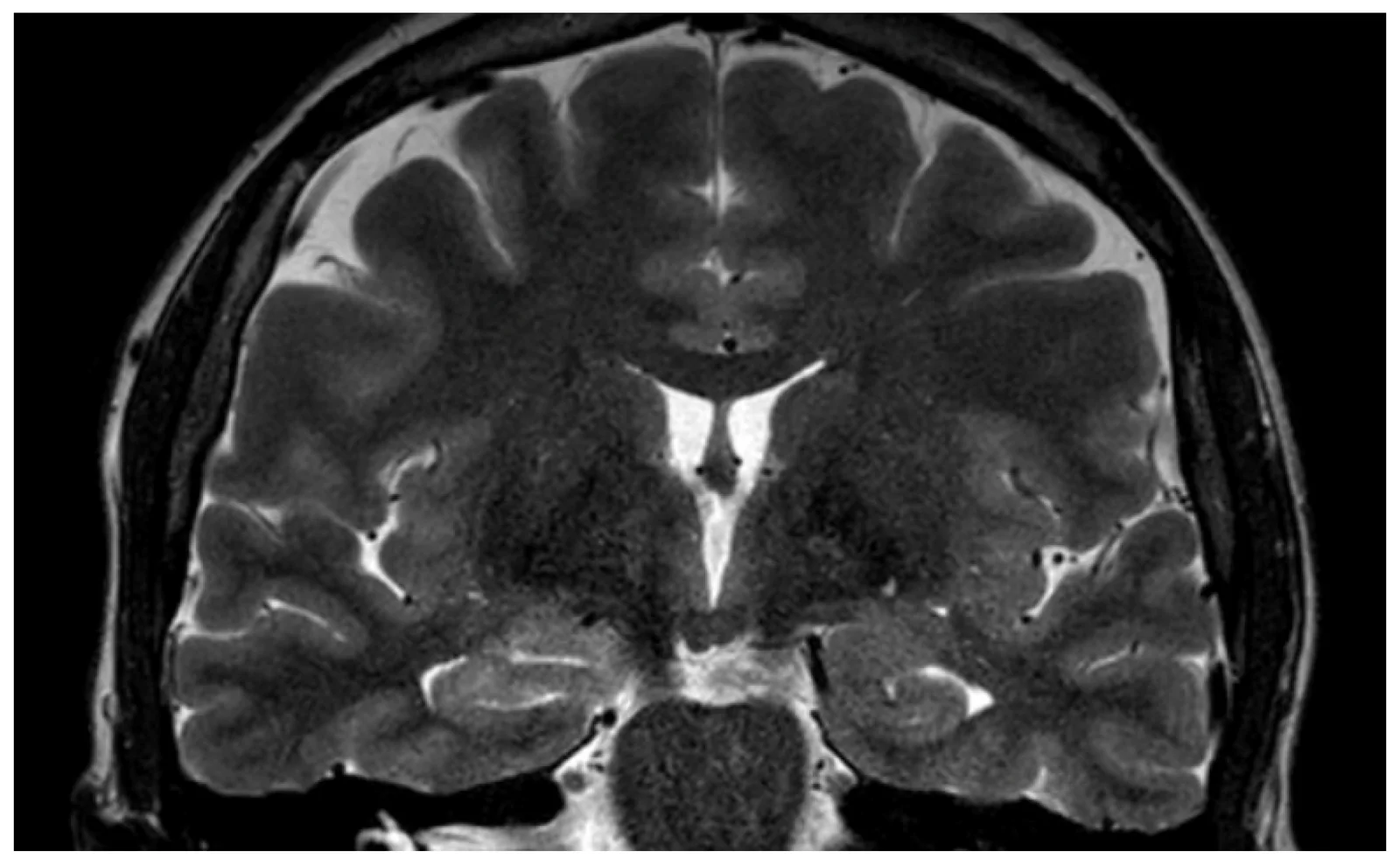
Coronal T2-weighted MRI through the medial temporal lobes demonstrating interval regression of the previously described right hippocampal lesion, with no clearly demarcated residual hyperintense focus visible on this sequence.

The patient reported her last menstrual period in September 2025, after which progestin therapy was discontinued. The most recent brain MRI, performed in March 2026, included coronal T2-weighted sections through the medial temporal lobes and bilateral hippocampal formations (Figure 3). Compared with previous examinations, the MRI showed absence or marked reduction in the previously described right hippocampal T2-hyperintense signal. Both hippocampi demonstrated overall preserved morphology, with no evident focal T2-hyperintense lesions and no significant structural distortion. These findings were considered compatible with lesion regression or residual chronic gliotic changes. The lesions of cerebral endometriotic foci can become gliotic following menopause.

Compared with the examination performed on 28 July 2020, the MRI examination performed on 20 March 2026 showed a reduction in the volume of the right hippocampus from 4.7 mL to 4.2 mL and a smaller reduction in the left hippocampal volume from 3.2 mL to 3.1 mL. Reductions were also observed in bilateral mesiotemporal structures, temporal lobes, and cerebellar cortex. These findings indicate longitudinal changes in whole-structure brain volumes but do not constitute a quantitative measurement of the focal hippocampal lesion. Image analysis was carried out using the “mdbrain” software (version 2.2) developed by Mediaire GmbH (Berlin, Germany), which is certified in accordance with European medical device regulations. The software is authorized for clinical use under the requirements of the European Commission and is designed for automated brain volumetric assessment. It analyzes native 3D T1-weighted MRI sequences to quantify volumes across multiple brain regions and lobes. The system incorporates a proprietary deep learning segmentation algorithm based on a U-Net architecture, enabling efficient and reliable volumetric evaluation. The software provides volumetric measurements for 42 distinct brain structures, including the hippocampus, and expresses results as percentiles relative to a large normative database of healthy individuals (n = 6371; age range 10–97 years). These comparisons are adjusted for age, sex, and total intracranial volume (ICV). Reported metrics include total brain volume (TBV), gray matter (GM), white matter (WM), and cortical gray matter (cGM).

Compared to the examination on 28 July 2020, the examination on 20 March 2026 reveals a global decrease in brain volume, predominantly in the white matter, with a reduction in the percentile of total brain volume (from 55.8% to 28.8%). A volumetric reduction is noted at the bilateral mesiotemporal level, including the hippocampus, parahippocampal gyrus, and entorhinal cortex. At the hippocampus level, the right side decreased in volume (4.7 mL → 4.2 mL) but remained in the upper percentiles (≈93%), and the left side showed a slight decrease (3.2 mL → 3.1 mL), located in the lower percentile (≈23%). Hippocampal asymmetry is maintained (right > left), with a tendency for bilateral reduction over time. Associated with this, a decrease in volume of the bilateral temporal lobes and a marked reduction in the volume of the cerebellar cortex are highlighted. The overall appearance is suggestive of a discreetly progressive cerebral atrophy process, with mesio-temporal lobes involvement, within limits partially within the normative intervals, and periodic volumetric monitoring.

To provide a clear overview of the chronological relationship between therapeutic interventions, clinical evolution, and neuroimaging findings, the patient’s complete clinical course is summarized in Table 1.

### Ethical Considerations

The clinical case was conducted in accordance with the principles outlined in the Declaration of Helsinki. Written informed consent was obtained from the patient for the use of her anonymized clinical data and MRI images for research and publication purposes. Institutional ethical board approval was also obtained (6068/19 May 2026).

## 3. Literature Review

A narrative literature review was conducted to identify published case reports describing patients diagnosed with cerebral endometriosis. Eight case reports were identified. The following electronic databases were searched: PubMed, Scopus, Google Scholar, and Web of Science. The search strategy included combinations of the following terms: “cerebral endometriosis,” “brain endometriosis,” “sites of cerebral endometriosis,” “diagnosis,” and “treatment.” Duplicate records were removed, and the reference lists of the selected case reports were manually screened. In addition, the bibliographies of relevant articles and review papers were cross-referenced to identify further eligible reports. A manual search of conference abstracts, posters, and additional databases was also performed to enhance the completeness of the review. No restrictions were applied regarding study design. The literature search was last updated in August 2026.

Thibodeau et al. [15] reported in 1987 the case of a 20-year-old woman with a 3-year history of intermittent focal headaches and a generalized seizure. Imaging revealed a cystic lesion in the right parietal lobe. The lesion was surgically removed, and histological examination confirmed EM.

In 1993, Ichida et al. [16] described a 31-year-old woman with cerebral endometriosis who presented with recurrent partial seizures occurring on the first day of menstruation. After surgical removal of the brain lesions, which confirmed EM, her symptoms were controlled with danazol therapy.

Sarma et al. [17] reported in 2004 the case of a 40-year-old woman who presented with gait disturbance and headache. CT identified a midline posterior fossa mass, while MRI demonstrated a multiloculated cystic lesion arising from the superior vermis, with a lobulated component projecting into the superior cerebellar cistern. During surgery, the cyst was found to contain “chocolate-colored fluid.” Histological examination confirmed endometriosis, and immunohistochemistry supported the presence of endometrial-type epithelium. The patient’s symptoms improved after surgery. She denied cyclic headaches or seizures and had no history of pelvic pain or infertility.

Vilos et al. [18] reported in 2011 the case of a woman with catamenial neurological signs and symptoms. MRI and CT revealed a circumscribed lesion in the left centrum semiovale. Her neurological symptoms resolved completely after treatment with a gonadotropin-releasing hormone agonist for three months, followed by laparoscopic bilateral oophorectomy. The temporal association between neurological symptoms and menstruation, together with symptom resolution after medical and surgical menopause, was considered highly suggestive of cerebral EM.

In 2018, Maniglio et al. [19] presented the case of a 39-year-old woman with a 3-year history of hallucinations associated with catamenial epilepsy who had cerebral hemosiderosis deposits in the globus pallidus. The presence of endometriotic tissue was confirmed by biopsy.

Meggyesy et al. [20] in 2020 described the case of a 39-year-old woman who had undergone multiple operations for infratentorial brain cysts complicated by chronic hydrocephalus. She had no history of menstruation-related neurological symptoms and developed premature amenorrhea at approximately 20 years of age. At 27 years, a fourth-ventricle cyst and syringomyelia extending to L1 were identified. She later developed progressive gait and speech disturbances. At 35 years of age, she experienced status epilepticus lasting 40 min. Brain MRI revealed multiple infratentorial cysts compressing the brainstem. Posterior fossa decompression was performed, and the cysts were opened and partially resected. Histological examination confirmed cerebellar endometriosis. Progestogen therapy was initiated but did not improve the patient’s condition. She died at 39 years of age from complications related to cyst recurrence and hydrocephalus.

Antonio et al. [21] reported in 2021 the case of a 44-year-old woman with surgically diagnosed ovarian EM and catamenial epilepsy attributed to cerebral EM. The condition was resistant to medical therapy, and hormonal treatment was contraindicated due to cerebral ischemic episodes.

Elefante et al. [22] reported in 2022 a case of presumed cerebral endometriosis involving the frontal lobe in a 50-year-old woman. This case was notable for the coexistence of neurological and psychiatric manifestations. The patient had a long history of atypical bipolar disorder features, including chronic mood instability, mixed episodes, excitatory interepisodic symptoms, and panic disorder for more than 25 years. Brain MRI revealed two focal lesions with a malacic center in the subcortical white matter of the left hemisphere: one in the anterior frontal region and another in the postero-inferior parietal region. These lesions were initially interpreted as postischemic. Hemosiderin deposits were also detected in the right hemisphere near the uncus and in the frontal subcortical region. After iatrogenic menopause, the patient’s gynecological and neurological symptoms remitted, whereas the psychiatric symptoms persisted. The authors considered the findings compatible with presumed cerebral EM.

The main clinical, radiological, diagnostic, therapeutic, and outcome characteristics of previously published cases of cerebral EM, together with the present case, are summarized in Table 2.

## 4. Discussion

EM most commonly affects women of reproductive age, particularly between 20 and 40 years, although it may occur from menarche to postmenopause, irrespective of race, ethnicity, or parity [23]. In the present case, neurological symptoms began at the age of 41. This is relevant because EM may remain clinically silent or minimally symptomatic for many years. Not all affected individuals report typical symptoms, and only a proportion of patients describe manifestations such as dysmenorrhea, dyspareunia, chronic pelvic pain, or infertility. Consequently, the diagnosis of EM is often delayed, with reported diagnostic intervals ranging from 5 to 12 years [24,25]. In our case, the absence of dysmenorrhea, dyspareunia, and cyclic pelvic pain made the diagnosis particularly challenging.

The risk factors for cerebral EM remain poorly defined because of the extreme rarity of this condition. A history of pelvic or ovarian EM may increase clinical suspicion, but cerebral involvement can also be considered in patients without typical pelvic symptoms. Therefore, the absence of known pelvic EM should not exclude the possibility of an endometriosis-related intracranial lesion, especially when neurological manifestations show a catamenial pattern.

EM is increasingly regarded not only as a localized pelvic disease but also as a systemic inflammatory disorder with potential neurological implications. Beyond direct infiltration of nerves or distant ectopic implantation, endometriosis may contribute to neuroinflammation, altered pain processing, autonomic dysfunction, and structural or functional brain changes [26]. Chronic inflammatory signaling and central sensitization have been proposed as mechanisms by which endometriosis may influence the central nervous system, even in the absence of direct brain invasion. Inflammatory mediators released by active endometriotic lesions may contribute to altered pain perception and may affect brain regions involved in pain modulation, emotion, memory, and cognition. Experimental and clinical studies have suggested associations between EM and changes in brain structure or function, including reduced gray matter volume in selected regions; however, true intracranial implantation of endometriotic tissue remains exceptionally rare [27].

EM involving the central nervous system is an unusual form of extrapelvic disease. Most reported neurological cases involve the spinal canal, conus medullaris, cauda equina, dura, spinal cord, or vertebral structures, and may present with back pain, radiculopathy, motor or sensory deficits, or symptoms fluctuating with the menstrual cycle [28,29]. In contrast, cerebral EM is much less frequently described. The present case is unusual because the lesion was localized to the right hippocampus, a site that, to our knowledge, has not been clearly reported in previous cases of cerebral endometriosis.

The hippocampus is located in the medial temporal lobe and plays a central role in memory consolidation, spatial orientation, emotional processing, and stress regulation. Lesions in this region may be associated with short-term memory impairment, difficulty forming new memories, behavioral or emotional changes, and temporal lobe epilepsy. In our patient, the hippocampal localization is consistent with the clinical presentation, which included epileptic seizures and episodes of short-term memory loss. The catamenial pattern of symptoms further supported a possible hormone-sensitive mechanism.

The clinical spectrum of cerebral EM is heterogeneous. Reported complications include seizures, focal neurological deficits, headaches, gait disturbances, hydrocephalus, psychiatric manifestations, and cognitive or behavioral symptoms. Involvement of hypothalamic or pituitary regions could theoretically affect endocrine and reproductive function, although this remains extremely rare. Psychiatric manifestations have also been discussed in association with cerebral endometriosis, including possible links with mood disorders, bipolar-spectrum symptoms, anxiety, and panic disorder [30,31]. Preclinical data suggest that EM may be associated with microglial activation and glial changes in brain regions involved in mood and pain regulation, providing a possible biological basis for anxiety and depression-like symptoms [32]. However, in clinical practice, psychiatric symptoms are non-specific, and causality cannot be inferred without careful multidisciplinary assessment.

Diagnosis of cerebral EM is difficult because imaging findings are not pathogno-monic and may resemble neoplastic, inflammatory, infectious, vascular, or developmental lesions. Although increasing awareness and improved imaging techniques have facilitated non-invasive diagnosis of EM in several anatomical sites, cerebral involvement remains particularly challenging.

In the present case, the right hippocampal abnormality should not be regarded as radiologically specific for cerebral endometriosis. Mesial temporal T2/FLAIR hyperintensity has a broad differential diagnosis, including peri-ictal abnormalities, hippocampal sclerosis, infectious or autoimmune limbic encephalitis, ischemic injury, vascular lesions, and neoplastic processes. This distinction is particularly relevant in the present patient because epilepsy preceded the imaging follow-up and seizure activity itself may produce transient hippocampal T2/FLAIR abnormalities, sometimes accompanied by diffusion restriction, swelling, or subsequent structural changes. Peri-ictal MRI abnormalities may be reversible and can therefore mimic an underlying mass lesion, emphasizing the importance of longitudinal imaging and electroclinical correlation.

An inflammatory or autoimmune process, particularly limbic encephalitis, also represents a theoretical alternative because autoimmune encephalitis may produce unilateral or bilateral medial temporal T2/FLAIR hyperintensity and may subsequently lead to hippocampal atrophy. In the present patient, however, the clinical course was not characterized by an acute or subacute encephalopathic syndrome, progressive cognitive impairment, or other typical manifestations that would strongly favor an autoimmune limbic process. Nevertheless, in the absence of a dedicated autoimmune work-up documented at the time of the original presentation, this possibility cannot be considered definitively excluded and is therefore retained as part of the differential diagnosis.

A low-grade hippocampal neoplasm was also a relevant consideration, particularly after the development of pseudonodular imaging abnormality in 2020. The absence of diffusion restriction, lack of a clearly progressive mass effect, and the absence of a metabolic pattern strongly suggestive of an aggressive infiltrative tumor on MRS provided some arguments against a high-grade neoplastic process. However, these findings do not exclude a low-grade glioma or other indolent neoplasm. Accordingly, the regression of the lesion during subsequent follow-up was considered more informative than any individual MRI characteristic, although radiological regression alone cannot establish an endometriotic etiology.

The T1-hyperintense and T2-hypointense components observed during the 2020 examination also warranted consideration of hemorrhagic or vascular pathology, including lesions containing hemosiderin or other blood products. Such signal characteristics may occur in a variety of vascular and hemorrhagic conditions and are not specific for cerebral endometriosis. Similarly, the residual or regressed hippocampal signal abnormality could theoretically represent chronic gliotic change, including gliosis secondary to previous seizure-related injury. Therefore, the presence of gliosis should not be interpreted as direct evidence of endometriotic implantation.

Taken together, the diagnosis in this patient was therefore based on a pattern of convergent, but individually non-specific, findings rather than on a pathognomonic MRI appearance. The most relevant supportive features were the reproducible catamenial pattern of seizures, the temporal evolution of the hippocampal abnormality, the lack of convincing evidence for aggressive tumor infiltration on MRS, the complete clinical response following progestogen therapy, and the marked regression or disappearance of the previously observed T2/FLAIR abnormality during longitudinal follow-up. Nevertheless, because histopathological confirmation was not obtained, the appropriate diagnostic designation remains presumed cerebral endometriosis, and alternative seizure-related, inflammatory, neoplastic, vascular, and gliotic explanations cannot be excluded with absolute certainty.

Familiarity with the diverse imaging patterns of EM across organ systems may help radiologists include EM in the differential diagnosis, even when imaging studies are not primarily performed for suspected endometriotic disease.

Ultrasonography is not suitable for evaluating intracranial lesions because the skull strongly limits ultrasound penetration. Brain MRI remains the preferred non-invasive modality. T1-weighted, T2-weighted, and FLAIR sequences provide important information regarding lesion morphology, signal characteristics, surrounding edema, gliotic change, hemorrhagic components, and interval evolution [33]. MRI may also assist in differentiating cerebral EM from tumors, vascular malformations, inflammatory lesions, or infectious processes. Nevertheless, as shown in our case, even advanced MRI may not establish the diagnosis with certainty. Its main value was in the longitudinal assessment of lesion evolution and treatment response.

3D-MRI may improve visualization of cortical and deep brain structures and can support lesion localization and follow-up. Accurate segmentation of brain lesions on MRI is increasingly important for diagnosis, monitoring, and treatment planning, particularly in tumor-like lesions [34,35]. However, manual segmentation remains time-consuming and subject to interobserver variability. In the present case, after imaging evaluation, the initial differential diagnosis included focal cortical dysplasia, infection, tumor infiltration, and hippocampal tumor, but MRI was essential for dynamic evaluation. Longitudinal automated volumetry provided additional information regarding structural brain changes over time. The right hippocampal volume decreased from 4.7 mL to 4.2 mL between 2020 and 2026; however, because the software quantified the entire anatomical hippocampus rather than the focal T2/FLAIR abnormality, this change cannot be interpreted as a quantitative measure of regression of the presumed endometriotic lesion. The concomitant reduction in left hippocampal volume and in other mesiotemporal, temporal, and cerebellar structures further argues against attributing the volumetric findings specifically to regression of a focal endometriotic lesion. Accordingly, automated volumetry was considered complementary and exploratory, whereas assessment of lesion evolution relied primarily on serial qualitative MRI comparison.

MRS was also performed. MRS may provide metabolic information, but its additional diagnostic value varies depending on lesion type, location, and technical quality [36]. In our case, MRS did not demonstrate a metabolic profile clearly suggestive of tumor-like infiltration. The absence of lactate and lipid–macromolecule peaks argued against necrosis or a high-grade gliomatous process, but the findings were not diagnostic. Therefore, MRS was useful mainly as an adjunctive tool in excluding some aggressive neoplastic features rather than confirming cerebral EM.

Histological confirmation remains the diagnostic gold standard for EM. However, brain biopsy or surgical excision is not always feasible because of procedural risks, lesion location, and potential neurological consequences. In the present case, biopsy was not performed because the lesion was located in the right hippocampus, a functionally important area associated with memory and seizure activity. Therefore, the diagnosis was based on the combination of catamenial neurological symptoms, imaging characteristics, exclusion of alternative diagnoses, therapeutic response to progestins and radiological regression during follow-up. The favorable clinical course and reduction in the hippocampal lesion after treatment with dienogest and other progestins strongly supported the diagnosis of presumed cerebral EM.

Several mechanisms may explain the development and persistence of endometriotic lesions at distant sites. Angiogenesis, extracellular matrix remodeling, inflammatory activation, and altered immune surveillance are considered important in the pathogenesis of EM. The formation of new vessels from pre-existing vasculature may support survival of ectopic endometrial tissue. In addition, invasion of ectopic tissue involves extracellular matrix degradation and altered expression of matrix metalloproteinases. Because statins have antiproliferative, antiangiogenic, antioxidant, anti-inflammatory, and matrix metalloproteinase-modulating properties, they have been proposed as potential therapeutic agents in endometriosis [37]. However, there is currently insufficient evidence to support their use in cerebral endometriosis, and we have no experience with this approach.

Management of cerebral EM is not standardized because of the very limited number of reported cases. Treatment must be individualized according to lesion location, symptom severity, diagnostic certainty, reproductive considerations, response to medical therapy, and surgical risk. Surgery may be considered when lesions cause mass effect, hydrocephalus, progressive neurological deficits, diagnostic uncertainty, or failure of conservative therapy. Surgical resection has been successful in selected cases, particularly when lesions were accessible and histological confirmation was obtained. However, neurosurgical intervention carries risks, including neurological injury, infection, incomplete resection, and recurrence. For this reason, non-surgical management may be preferable when symptoms and imaging findings respond to hormonal suppression.

Hormonal therapy represents a rational therapeutic approach because EM is estrogen-dependent and often progesterone-responsive. Progestins, including dienogest, are widely used in the management of EM and are recommended in several clinical guidelines [38,39]. Dienogest is a fourth-generation progestin with high selectivity for progesterone receptors and antiestrogenic, anti-inflammatory, and anti-proliferative effects on endometriotic tissue. It is generally considered effective and well-tolerated for long-term treatment of EM [40,41]. In a previously published case of cerebral EM associated with catamenial epilepsy, complete remission was reported after dienogest therapy [20]. Similarly, in our patient, progestin-based treatment was associated with complete resolution of neurological symptoms, discontinuation of antiepileptic medication, and regression of the right hippocampal lesion on follow-up MRI.

The response to progestin therapy in our case is clinically important. The patient initially received antiepileptic treatment, including levetiracetam and lamotrigine, but seizure control remained suboptimal. After recognition of the catamenial pattern and initiation of hormonal therapy with desogestrel and dienogest, neurological symptoms resolved completely. This temporal association suggests that the seizures were at least partly hormone-sensitive and possibly related to presumed cerebral EM. However, because this is a single case without histological confirmation, causality cannot be definitively proven.

Although dienogest may be preferable to GnRH agonists or surgery in selected patients, long-term treatment requires monitoring. Prolonged dienogest use is usually well tolerated, but adverse effects may include irregular uterine bleeding, breast discomfort, acne, weight changes, mood symptoms, and hypoestrogenic effects. A particular concern is reduced bone mineral density (BMD), especially during extended treatment [42]. Predictive factors for BMD reduction are not fully established [43], although endogenous estradiol levels during dienogest therapy may have potential predictive value [44]. Kim et al. reported significant decreases in lumbar spine and femoral neck BMD after three years of dienogest treatment, with the greatest loss occurring during the first year [45]. Therefore, patients receiving long-term dienogest should be monitored clinically, and BMD assessment by dual-energy X-ray absorptiometry (DEXA) should be considered. Preventive strategies, including vitamin D, calcium or mineral supplementation, lifestyle measures, and individualized endocrine evaluation, may be appropriate. We recommended that our patient undergo a DEXA scan to assess BMD.

Supportive management is also important. Sleep disturbance, chronic stress, pain, anxiety, and mood symptoms may worsen neurological and gynecological outcomes. In our case, zopiclone was prescribed for severe insomnia. Although lifestyle interventions cannot replace medical therapy, sleep hygiene, stress reduction, psychological support, and treatment of comorbid mood or anxiety symptoms may improve overall quality of life. Cognitive-behavioral therapy may be useful in patients with chronic pain, sleep disturbance, or emotional distress.

Follow-up should be individualized. Regular neurological assessment, symptom diaries, and menstrual-cycle correlation may help detect recurrence or progression. Serial MRI is useful for monitoring lesion stability or regression and assessing treatment response. Some guidelines and expert recommendations suggest periodic imaging surveillance in complex EM, although the optimal interval for cerebral EM is unknown [46]. In practice, the frequency of follow-up imaging should be guided by symptom severity, lesion location, treatment response, and risk of progression.

This case has several limitations. The diagnosis was not histologically confirmed because biopsy of the hippocampal lesion was considered high-risk. Moreover, cerebral EM is extremely rare, and its imaging findings are nonspecific. An additional limitation concerns the electrophysiological assessment. Although routine interictal EEG showed no epileptiform abnormalities, neither ictal EEG nor prolonged video-EEG monitoring was available. Consequently, seizure classification relied primarily on the clinical semiology reported by the patient and the temporal relationship documented in the seizure–menstrual diary. While a normal interictal EEG does not exclude epilepsy, the absence of electroclinical documentation of the seizures limits diagnostic certainty. Nevertheless, the catamenial seizure pattern, the lack of clear MRS evidence of tumor-like infiltration, regression of the lesion on follow-up MRI, and complete resolution of symptoms after progestin therapy collectively support the diagnosis of presumed cerebral EM.

## 5. Conclusions

Cerebral EM is an exceptionally rare condition that requires careful clinical, radiological, and multidisciplinary evaluation. In women presenting with periodic or catamenial neurological symptoms, particularly seizures, EM should be considered in the differential diagnosis, even in the absence of typical pelvic symptoms. MRI is the main non-invasive imaging tool for identifying intracranial lesions and monitoring their evolution during follow-up. In the present case, recognition of the menstrual pattern of symptoms, consideration of alternative diagnoses without convincing evidence for several competing etiologies, and radiological regression after progestin therapy collectively supported the diagnosis of presumed cerebral EM. Hormonal treatment may represent an effective conservative option in selected patients, although management should be individualized. Collaboration among gynecologists, neurologists, neuroradiologists, neurosurgeons, and mental health specialists is essential for comprehensive care and long-term monitoring.

## Figures and Tables

**Table 1:** Timeline of treatment, clinical course, and neuroimaging findings.

Period	Treatment	Clinical Manifestations/Course	Imaging Findings
**February 2019–August 2019**	No antiepileptic treatment	Onset of recurrent epileptic seizures in February 2019, persisting for 6 months until neurological evaluation.	No brain imaging was performed at symptom onset.
**August 2019–February 2020**	Levetiracetam 500 mg twice daily	Antiepileptic treatment was initiated; however, seizures persisted after 6 months of therapy.	August 2019 MRI: diffuse right hippocampal cortico-subcortical lesion involving the head, body, and tail of the hippocampus; mildly hypointense on T1-weighted images and hyperintense on T2/FLAIR images, without contrast enhancement.
**February 2020–May 2020**	Levetiracetam plus lamotrigine	Combination therapy was administered for 3 months.	Follow-up MRI in 2020: persistent right hippocampal lesion with an 11 × 10 mm pseudonodular contrast-enhancing area in its posterior portion and an associated T2-hypointense nodular area.
**May 2020–August 2020**	Lamotrigine monotherapy	Lamotrigine monotherapy was continued for another 3 months; seizure frequency subsequently increased.	MRS: decreased right-sided N-acetylaspartate, Cho/NAA ratio of 0.8, and Cho/Cr ratio of 0.9, without lipid–macromolecule or lactate peaks. The findings were inconclusive and did not provide clear evidence of tumor-like infiltration.
**August 2020–February 2021**	Levetiracetam	Because of the increased seizure frequency, the patient was switched back to levetiracetam for another 6 months.	No additional relevant imaging changes were documented during this period.
**February 2021–September 2025**	Hormonal therapy: Cerazette (desogestrel) alternating with dienogest in 6-month cycles; levetiracetam was administered concomitantly during the first 3 months	Hormonal therapy was initiated after recognition of the catamenial seizure pattern. Antiepileptic therapy was subsequently discontinued, followed by complete resolution of the neurological symptoms.	No interval MRI examination was reported during the hormonal treatment period.
**September 2025–March 2026**	Progestin therapy discontinued after the last menstrual period	Complete clinical remission was maintained after discontinuation of hormonal and antiepileptic treatment.	March 2026 MRI: absence or marked reduction in the previously described right hippocampal T2/FLAIR hyperintensity. Both hippocampi showed preserved morphology, without significant structural distortion.
**April 2026**	No active hormonal or antiepileptic treatment	Neurological symptoms remained resolved.	MRI volumetric analysis demonstrated longitudinal changes in whole-structure brain volume, including a reduction in right hippocampal from 4.7 mL to 4.2 mL and a slight reduction in left hippocampal volume from 3.2 mL to 3.1 m. These measurements reflect whole-structure volumetric changes and do not constitute quantitative assessment of the focal hippocampal lesion.

**Table 2:** Published cases of cerebral endometriosis, including the present case.

Author, Year	Age	Lesion Site	Main Clinical Presentation	Catamenial Pattern	Pelvic/Ovarian Endometriosis	Diagnosis/Confirmation	Treatment	Outcome
Thibodeau et al. 1987 [15]	20	Right parietal lobe	Intermittent focal headaches for 3 years; generalized seizure	Not clearly reported	Not reported	Surgical excision; histology confirmed endometriosis	Surgery	Not fully reported; diagnosis confirmed histologically
Ichida et al. 1993 [16]	31	Cerebral lesions; exact site not specified in current summary	Recurrent partial seizures occurring on the first day of menstruation	Yes	Not reported	Surgical removal of brain lesions; cerebral endometriosis diagnosed	Surgery followed by danazol	Symptoms controlled after treatment
Sarma et al. 2004 [17]	40	Posterior fossa; superior vermis/cerebellar region	Gait disturbance and headache	No; patient denied cyclic headaches or seizures	No history of pelvic pain or infertility reported	Surgery revealed cyst with “chocolate-colored fluid”; histology and immunohistochemistry confirmed endometriosis	Surgical excision	Clinical improvement after surgery
Vilos et al. 2011 [18]	41	Left centrum semiovale	Catamenial neurological signs and symptoms	Yes	Presumed/suggestive; details not specified	MRI and CT showed circumscribed brain lesion; diagnosis presumed based on menstrual association and response to induced menopause	GnRH agonist for 3 months followed by laparoscopic bilateral oophorectomy	Complete resolution of neurological symptoms
Maniglio et al. 2018 [19]	39	Cerebral endometriosis exact site not found	Hallucinations in the context of catamenial epilepsy	Yes	Endometriotic tissue confirmed by biopsy	Biopsy confirmed endometriotic tissue	Dienogest/progestin-based therapy	Complete remission reported by authors
Meggyesy et al. 2020 [20]	39	Infratentorial region/cerebellum; fourth ventricle cysts with brainstem compression	Progressive gait and speech deficits; status epilepticus; chronic hydrocephalus	No menstruation-related neurological symptoms reported; premature amenorrhea around age 20	Not specified	Posterior fossa decompression and partial cyst resection; histology confirmed cerebellar endometriosis	Surgery followed by progestogen therapy	No clinical improvement; death at age 39 due to cyst recurrence and hydrocephalus
Antonio et al. 2021 [21]	44	Cerebral endometriosis; exact site not specified	Catamenial epilepsy resistant to medical therapy	Yes	Surgically diagnosed ovarian endometriosis	Diagnosis described as cerebral endometriosis related to catamenial epilepsy; details not specified	Medical therapy attempted; hormonal therapy contraindicated due to cerebral ischemic episodes	Medical therapy-resistant case
Elefante et al. 2022 [22]	50	Frontal lobe/subcortical white matter; lesions in left anterior frontal and postero-inferior parietal regions; hemosiderin deposits near right uncus and frontal subcortical region	Neurological and psychiatric manifestations; long history of mood instability, mixed episodes, excitatory interepisodic symptoms, and panic disorder	Neurological and gynecological symptoms improved after iatrogenic menopause; psychiatric symptoms less clearly related	Gynecological history suggestive;	Presumed cerebral endometriosis based on clinical, imaging, and gynecological assessment	Iatrogenic menopause/hormonal suppression	Gynecological and neurological symptoms remitted; psychiatric symptoms persisted
Present case	48	Right hippocampus, posterior hippocampal region	Catamenial epilepsy with short-term memory loss; severe insomnia later reported	Yes	No dysmenorrhea, dyspareunia, or cyclic pelvic pain; no clinical symptoms suggestive of pelvic endometriosis	MRI showed right hippocampal lesion with interval change; MRS inconclusive; diagnosis presumed based on catamenial symptoms, imaging evolution, and response to progestins	Levetiracetam and lamotrigine initially; later, Cerazette alternated with dienogest; zopiclone for insomnia; antiepileptic therapy discontinued	Complete resolution of neurological symptoms reported after progestin therapy; follow-up MRI showed absence/marked reduction in previous right hippocampal T2/FLAIR hyperintensity

Abbreviations: CT, computed tomography; GnRH, gonadotropin-releasing hormone; MRI, magnetic resonance imaging; MRS, magnetic resonance spectroscopy.

## Data Availability

The raw data supporting the conclusions of this article will be made available by the authors on request.
